# Photooxidation of A2E by Blue Light Regulates Heme Oxygenase 1 Expression via NF-κB and Lysine Methyltransferase 2A in ARPE-19 Cells

**DOI:** 10.3390/life12111698

**Published:** 2022-10-25

**Authors:** Chae Young Shin, Kwang Won Jeong

**Affiliations:** Gachon Research Institute of Pharmaceutical Sciences, College of Pharmacy, Gachon University, 191 Hambakmoero, Yeonsu-gu, Incheon 21936, Korea

**Keywords:** retinal pigment epithelium, N-retinylidene-N-retinylethanolamine, blue light, NF-κB, heme oxygenase 1, lysine methyltransferase 2A

## Abstract

**Background:** N-retinylidene-N-retinylethanolamine (A2E) is a component of drusen that accumulates in retinal cells and induces oxidative stress through photooxidation, such as blue light (BL). We found that the heme oxygenase 1 (*HMOX1*) gene responds sensitively to photooxidation by the BL of A2E in retinal pigment epithelial (RPE) cells, and we sought to identify the transcription factors and coactivators involved in the upregulation of *HMOX1* by A2E and BL. **Methods:** A2E-laden human RPE cells (ARPE-19) were exposed to BL (430 nm). RNA sequencing was performed to identify genes responsive to BL exposure. Chromatin immunoprecipitation and RT-qPCR were performed to determine the regulation of *HMOX1* transcription. Clinical transcriptome data were used to evaluate *HMOX1* expression in patients with age-related macular degeneration (AMD). **Results:** In ARPE-19 cells, the expression of *HMOX1*, one of the NF-κB target genes, was significantly increased by A2E and BL. The binding of RELA and RNA polymerase II to the promoter region of *HMOX1* was significantly increased by A2E and BL. Lysine methyltransferase 2A (MLL1) plays an important role in H3K4me3 methylation, NF-κB recruitment, chromatin remodeling at the *HMOX1* promoter, and, subsequently, *HMOX1* expression. The retinal tissues of patients with late-stage AMD showed significantly increased expression of *HMOX1* compared to normal retinal tissues. In addition, the expression levels of *MLL1* and *HMOX1* in retinal tissues were correlated. **Conclusions:** Taken together, our results suggest that BL induces *HMOX1* expression by activating NF-κB and MLL1 in RPE cells.

## 1. Introduction

Nuclear factor (NF)- κB, a transcription factor that plays a key role in inflammation and apoptosis, regulates oxidative stress and DNA damage under various in vitro and in vivo experimental conditions. Structurally, NF-κB contains p50, p52, p65 (RELA), c-Rel (REL), and RELB, and a combination of these proteins creates homodimers and heterodimers. The mechanism of NF-κB activation has been well documented [1]. In most cells, NF-κB is present in the cytoplasm in an inactive form prior to the release of inhibitory IκB protein by phosphorylation. In addition to stimulatory factors, such as tumor necrosis factor (TNF)α, continuous exposure to ultraviolet (UV) light can activate the NF-κB pathway in human keratinocytes [2,3,4,5,6]. Later, it was found that short-wavelength visible light (e.g., blue light, BL) irradiation in mammalian cells also induces cellular damage, including mitochondrial DNA damage, through reactive oxygen species (ROS), such as hydroxyl radicals, superoxide anions, and singlet oxygen [7,8,9]. The ROS-induced cellular stress mechanism crosstalks with NF-κB signaling [10,11]. BL irradiation in human keratinocytes increases the production of ROS and TNFα through increased calcium influx via transient receptor potential cation channel subfamily V member 1 (TRPV1) activation [12]. 

Melanin and lipofuscin are sensitizers for short-wavelength visible light in pigmented cells, such as retinal pigment epithelial (RPE) cells. When BL with a wavelength of 430 nm is irradiated to human retinal pigment epithelial cells (ARPE-19), in which pyridinium bisretinoid A2E, an aging-related fluorophore, accumulates, DNA damage occurs in the form of oxidation base modification [13]. A2E is produced in the RPE due to the abnormal formation of ethanolamine condensation products of vitamin A, a major effector of the visual cycle [14,15,16]. In normal cases, it exists at a very low level in RPE cells. However, when an abnormal accumulation of A2E continues, various side effects are observed, such as the alkalinization of lysosomes and the secretion of pro-apoptotic proteins from the mitochondria [17,18]. More importantly, A2E is a major photosensitizer for BL in RPE cells [19]. Photooxidized A2E is converted into A2E-epoxide, which produces ROS, such as singlet oxygen [20,21]. A recent study reported the effect of A2E photooxidation on autophagy. Autophagy, a type of intracellular recycling process, forms the autophagosome, which then removes intracellular components through lysosomes and fusion [22]. Photooxidation of A2E dramatically inhibits lysosome function and blocks autophagolysosome formation, thereby reducing autophagic flux [23]. 

In recent decades, the effect of photooxidation of A2E by BL on RPE cells has been studied from various perspectives. In particular, the importance of NF-κB pathway activation, as a result of genome-wide transcription analysis, was highlighted in cytotoxic levels of A2E and BL exposure [24], and in models of prolonged low, non-cytotoxic A2E and BL exposure [25]. In addition, based on the results of these recent studies, efforts have been made to reduce the cytotoxicity caused by A2E and BL in retinal cells by removing ROS or blocking the NF-κB pathway [26,27,28]. However, the detailed expression mechanisms of individual genes that can be directly involved in the oxidative stress and apoptosis of RPE cells after NF-κB activation by A2E+BL are still unclear. Moreover, our understanding of the genes identified in models in vitro, and their correlations in clinical patients, is very limited. This study identified the *HMOX1* gene, which responds sensitively to the photooxidation process by the BL of A2E in RPE cells. We found that the NF-κB activation mediates the *HMOX1* by BL, and that this process is assisted by the co-regulator histone lysine methyltransferase 2A (MLL1). Finally, the correlation between the expression of *HMOX1* and its coactivator MLL1 in patients with age-related macular degeneration (AMD) was investigated, suggesting the possibility of future clinical applications.

## 2. Materials and Methods

### 2.1. Cell Culture and Reagents

Human retinal pigment epithelial cells (ARPE-19), purchased from the American Type Culture Collection (Manassas, VA, USA), were cultured in Dulbecco’s Modified Eagle’s Medium (DMEM)/F-12 medium (DMEM F-12; WELGENE, Gyeongsan, Korea), supplemented with 10% fetal bovine serum at 37 °C in a 5% CO_2_ environment. A2E was purchased from Key Synthesis LLC (Philadelphia, PA, USA).

### 2.2. In Vitro RPE Damage Model Induced by A2E and BL

An in vitro RPE damage model induced by A2E and BL was established, as previously described [24]. ARPE-19 cells were plated in a 6-well plate at a density of 2 × 10^4^ cells/well, and then treated with A2E (25 μM) three times at 48-h intervals. After 24 h of the last A2E treatment, the cells were irradiated with BL 3.5 cm away from the LED (430 nm, 8000 lux) for 30 min. After an additional 24 h of incubation, the total RNA and lysates were prepared, as previously described [28]. 

### 2.3. RNA-Sequencing 

The isolated total RNA was isolated using TRIzol reagent (Invitrogen, Carlsbad, CA, USA), and further purified using a RNeasy Mini Kit (QIAGEN, Hilden, Germany). RNA-seq was performed as previously described [24]. Briefly, the quality of purified RNA was assessed using an Agilent 2100 bioanalyzer RNA kit (Agilent, Santa Clara, CA, USA). The mRNA-seq library was prepared using a TruSeq Stranded mRNA kit (Illumina, San Diego, CA, USA) and sequenced using a NextSeq500 sequencer (Agilent). Genes exhibiting an absolute fold change (FC) of at least 2.0 and a *Q*-value < 0.05 between groups were considered differentially expressed. Differential expression datasets were subjected to KEGG 2021 and MSigDB hallmark analyses using EnrichR [29,30].

### 2.4. Quantitative Reverse Transcription Polymerase Chain Reaction (RT-qPCR)

Total RNA from ARPE-19 cells was isolated using TRIzol reagent (Invitrogen). The mRNA was reverse transcribed using an iScript cDNA synthesis kit (Bio-Rad Laboratories, Hercules, CA, USA). RT-qPCR was performed on a Roche LightCycler^®^ 480 II system using SYBR Green I Master Mix (Roche, Basel, Switzerland). Primer sequences used for RT-qPCR are listed in Supplementary Table S1. All mRNA expression levels were normalized to the *18S* rRNA levels.

### 2.5. Chromatin Immunoprecipitation 

The (chromatin immunoprecipitation) ChIP assay was performed, as previously described [31]. Cells were crosslinked with 1% formaldehyde for 30 min and then sonicated to shear the chromatin fragments. Sonicated chromatin was immunoprecipitated using an antibody overnight at 4 °C. The antibodies used for the ChIP assay were anti-Pol II (Millipore, Burlington, MA, USA), anti-p65 (Abcam, San Francisco, CA, USA), anti-MLL1 (Abgent, San Diego, CA, USA), and anti-H3K4me3 (Active motif, Carlsbad, CA, USA). After reverse crosslinking by heating, RT-qPCR was performed on the purified DNA fragment using a LightCycler^®^ 480 II system and SYBR Green I Master (Roche). The results are shown as the mean ± standard deviation (SD), expressed as a percentage of input chromatin. The primers used to amplify the *HMOX1* promoter or enhancer region are listed in Supplementary Table S2.

### 2.6. Formaldehyde-Assisted Isolation of Regulatory Elements (FAIRE)-qPCR

FAIRE-qPCR was performed, as previously described [31]. ARPE-19 cells were cross-linked with 1% formaldehyde, and then sonicated, to shear the chromatin fragments. Results are shown as the mean ± SD. Input DNA is expressed as a percentage of input chromatin. The sequences of the FAIRE-qPCR primers were identical to those used for the ChIP-qPCR analysis. 

### 2.7. RNA Interference and Transfection

ARPE-19 cells (2 × 10^4^ cells/well in a 6-well plate) were transfected with siRNA (siMLL1 or siMLL2), or non-specific siRNA (siNS), targeting the mRNA of *MLL1* and *MLL2*, according to the manufacturer’s protocol, using Oligofectamine (Invitrogen, Carlsbad, CA, USA). Additionally, independent siRNAs (siMLL1(1) and siMLL1(2)) acting on different sites of the *MLL1* mRNA were used to rule out the possibility of off-target effects. The siRNA sequences used in the experiments are listed in Supplementary Table S2.

### 2.8. Western Blotting

Western blot analysis was performed using anti-MLL1 (Abcam) and anti-β-actin (Santa Cruz, CA, USA) antibodies. Cell lysates were prepared using radioimmunoprecipitation assay buffer (RIPA) (50 mM Tris-HCl pH 8.0, 150 mM NaCl, 2 mM ethylenediaminetetraacetic acid, 1% sodium dodecyl sulfate (SDS), 1% sodium deoxycholate, and 1% NP- 40). Total cell lysates were ultrasonically dispersed for 10 min and centrifuged at 10,000× *g* for 20 min at 4 °C. The proteins were transferred to Immun-Blot polyvinylidene difluoride (PVDF) membranes (Bio-Rad, Hercules, CA, USA). Primary antibodies were used at a dilution of 1:1000 and then further incubated at 4 °C overnight. The membranes were then incubated with horseradish peroxidase (HRP)-labeled anti-rabbit second antibodies for 2 h at 1:5000 diluting in blocking solution. Expression levels were quantified by densitometry, followed by normalization to the β-actin.

### 2.9. Statistical Analysis

All statistical data were analyzed using GraphPad Prism 8.0.2 (GraphPad, San Diego, CA, USA), and are expressed as mean ± standard deviation (SD). Statistically significant differences between groups were determined using one-way analysis of variance (ANOVA), and were considered statistically significant at *p* < 0.05.

## 3. Results

### 3.1. Activation of TNFα Signaling Pathway by A2E and Blue Light in ARPE-19 Cells

**Figure 1:**
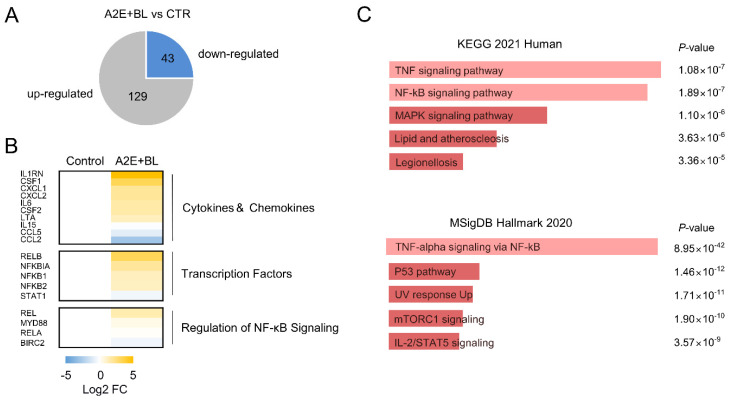
Activation of TNFα signaling by A2E and BL in ARPE-19 cells. (**A**) RNA-seq of in AREP-19 cells treated with A2E and BL (A2E+BL). The number of DEGs (129 upregulated and 43 downregulated) were shown in the pie chart (|FC| ≥ 2.0, *Q* < 0.05). (**B**) Heat map of genes responsible for cytokines and chemokines, transcription factors, and regulation of TNFα signaling. (**C**) Bar charts of potential signaling pathways generated using DEGs by A2E+BL in ARPE-19 cells. Pathway analyses were performed using KEGG and MSigDB.

ARPE-19 cells were treated thrice with A2E (25 µM) at 2-day intervals to induce intracellular A2E accumulation, followed by exposure to BL. RNA-seq was performed using the above cells, and differentially expressed genes were identified. Compared to the untreated control group, 172 (129 upregulated and 43 downregulated) genes were differentially expressed (|FC| ≥ 2.0, Q < 0.05) by A2E and BL (Figure 1A). No genes exhibited statistically significant expression changes after treatment with A2E alone or BL alone. Notably, the expression of cytokines and chemokine genes was significantly increased by A2E and BL (Figure 1B). Two different pathway analysis methods (KEGG and MSigDB) were performed to identify the biological pathway influenced by A2E and BL in ARPE-19 cells [29,30]. KEGG analysis revealed that differentially expressed genes (DEGs) by A2E and BL were enriched in the TNFα (*p* = 1.08 × 10^−7^) and NF-κB signaling pathways (*p* = 1.89 × 10^−7^, Figure 1C, upper panel). Similarly, in the MSigDB analysis, DEGs were enriched in TNFα signaling via the NF-κB pathway (*p* = 8.95 × 10^−42^, Figure 1C, lower panel). These results suggest that A2E and BL characteristically and remarkably activate the TNFα signaling pathway in ARPE-19 cells.

### 3.2. Blue Light Activates RELA-Mediated Transcription in ARPE-19 Cells

**Figure 2:**
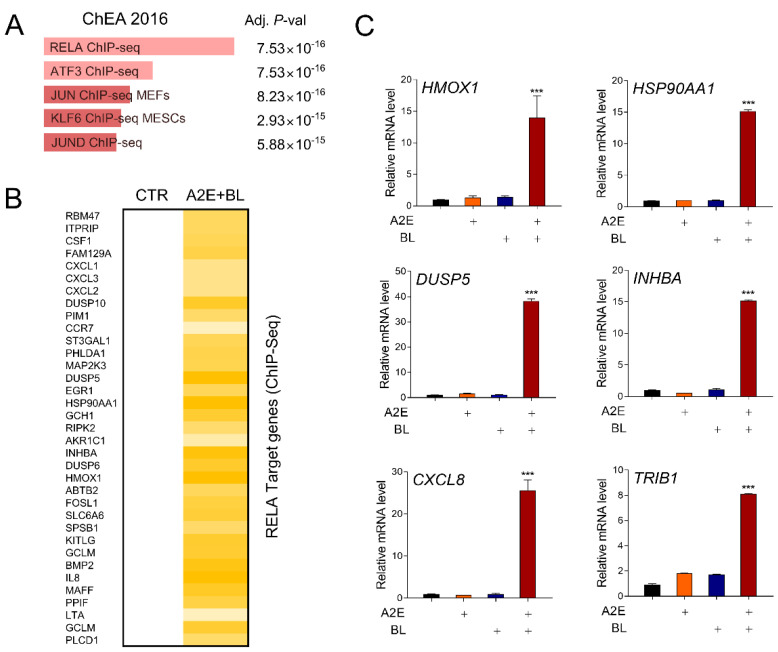
Blue light (BL) activates the NF-κB signaling pathway in ARPE-19 cells. (**A**) In silico analysis of upstream transcription factors regulating the expression of DEGs by A2E+BL in ARPE-19 cells. The length of the bars represents the combined score from the Fisher exact test. The adjusted *p*-value represents the statistical significance for specific terms. (**B**) The effect of A2E+BL on the expression of a set of RELA target genes in ARPE-19 cells. (**C**) Validation of RNA-seq results by RT-qPCR (reverse transcript-quantitative polymerase chain reaction) analysis showing the mRNA levels of RELA target genes in ARPE-19 cells treated (+marked) with A2E, BL, or A2E+BL vs. non-treated control. The mRNA levels were normalized to that of *18S* rRNA. Each value represents mean ± SD (*n* = 3), *** *p* < 0.001. *HMOX1*, Heme Oxygenase 1; *HSP90AA1*, heat shock protein 90 alpha family class A member 1; *DUSP5*, dual specificity phosphatase 5; *INHBA*, inhibin subunit beta A; *CXCL8*, C-X-C motif chemokine ligand 8; *TRIB1*, tribbles pseudokinase 1.

Next, we predicted the regulatory factors of these DEGs using in silico analysis. RELA (p65) was predicted as the most statistically significant upstream transcriptional regulator (Figure 2A). The NF-κB transcription factor exhibits various functions depending on the combination of various homo- and heterodimers. In particular, a heterodimer containing RELA (p65) and c-Rel acts as a transcriptional activator [32]. Indeed, in addition to the genes in the TNFα signaling pathway presented in Figure 1B, the expression levels of many previously reported RELA target genes were upregulated by A2E and BL treatment (Figure 2B). To verify the results of the in silico analysis, the effect of A2E+BL on a representative RELA target gene was confirmed using RT-qPCR in ARPE-19 cells. All six genes (*HMOX1*, *HSP90AA1*, *DUSP5*, *INHBA*, *CXCL8*, and *TRIB1*) confirmed in the experiment showed no change in their expression levels after A2E or BL treatment, but they did show a significant increase in expression with A2E+BL treatment (Figure 2C). These results suggest that photooxidation of A2E by BL activates NF-κB-mediated transcription in ARPE-19 cells.

### 3.3. Involvement of MLL1 in HMOX1 Gene Expression Induced by A2E and Blue Light

**Figure 3:**
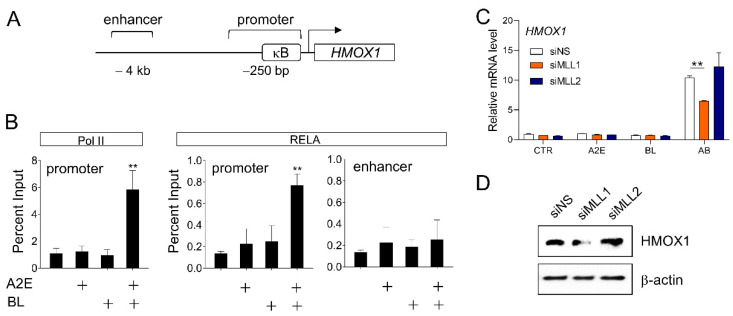
Recruitment of NF-κB and effect of MLL1 in *HMOX1* gene expression induced by A2E and blue light (BL). (**A**) The location of NF-κB binding sites in the *HMOX1* promoter region. (**B**) ChIP (chromatin immunoprecipitation) assay showing the recruitment of RELA (p65) and RNA Pol II to the *HMOX1* promoter region after the treatment (+ marked) of A2E, BL, or A2E+BL. The enhancer region lacking an NF-κB binding site was used as a negative control. The occupancies of RELA and Pol II were calculated as a percentage of input. Each value represents mean ± SD, ** *p* < 0.01). (**C**) ARPE-19 cells were transfected with siNS (non-specific siRNA), siRNA targeting *MLL1* (siMLL1), or siRNA targeting *MLL2* mRNA (siMLL2). After 72 h of transfection, total RNA was extracted by TRIzol and analyzed by RT-qPCR, and the mRNA levels of *HMOX1* were normalized to that of *18S* rRNA. Each value represents mean ± SD (*n* = 3), ** *p* < 0.01. (**D**) Protein levels of HMOX1 in MLL1 or MLL2-depleted ARPE-19 cells measured by Western blotting.

Among the RELA target genes identified earlier, HMOX1 drew our attention because HMOX1 is a representative oxidative stress response gene [33], RPE cells are one of the representative cells continuously exposed to various light sources including BL [34], and BL induces oxidative stress that can potentially induce apoptosis of retinal cells [35]. Therefore, we conducted an in-depth study on the expression mechanism of *HMOX1* in response to oxidative stress induced by A2E and BL in retinal cells. Chromatin immunoprecipitation was performed to confirm that A2E and BL regulate the expression of *HMOX1* at the transcriptional level. The *HMOX1* gene contains a NF-κB binding site in the promoter region, and the binding of RELA protein to the promoter region was significantly increased by A2E+BL (Figure 3A,B). Concomitantly, the binding of RNA Pol II to the promoter region was also significantly increased. In contrast, no significant change in the binding of RELA was observed in the enhancer region of *HMOX1*, which was used as a negative control. 

Although BL belongs to the visible light region, it has a short wavelength and high energy; therefore, it has photoactivity comparable to that of UV, supported by reports of BL-induced DNA damage in RPE cells [13,36,37]. The activated DNA damage response (DDR) induces inflammation by secreting a series of cytokines known as senescence-associated secretory phenotype (SASP) [38]. SASP expression is dependent on various transcription factors (e.g., NF-κB and C/EBPβ) [39,40], kinases (e.g., p38 MAPK and protein kinase D1) [41,42]. In particular, it is known that MLL1, a transcriptional regulator and histone H3K4 methyltransferase, is linked to DDR and plays an important role in SASP expression. In addition, the role of MLL1 in NF-κB target gene expression by TNFα stimulation in MEF cells has been reported [43]. siRNA was prepared to examine whether MLL1 is involved in *HMOX1* expression by A2E+BL, an ARPE-19 cell line in which MLL1 was depleted. In ARPE-19 cells treated with non-specific siRNA, *HMOX1* expression by A2E+BL was significantly increased. In contrast, MLL1-depleted ARPE-19 cells showed significantly reduced *HMOX1* expression. MLL2, another SET domain family histone H3K4 methyltransferase similar to MLL1, had no significant effect on the expression of *HMOX1* induced by A2E+BL (Figure 3C,D). 

These results suggest that NF-κB binds to the *HMOX1* gene promoter region upon A2E and BL stimuli to regulate the expression of the *HMOX1* at the transcriptional level, and that MLL1 plays an important role in this process.

### 3.4. MLL1 co-Activates NF-κB-Mediated HMOX1 Gene Expression in ARPE-19 Cells

**Figure 4:**
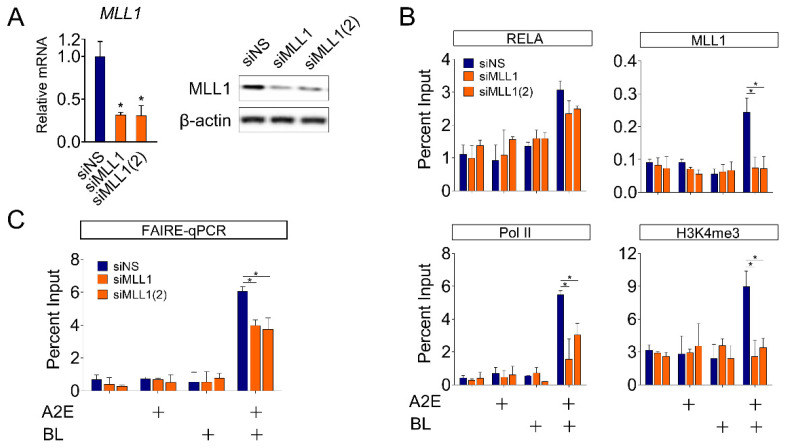
Effect of MLL1 on NF-κB-mediated *HMOX1* gene expression in ARPE-19 cells. (**A**) Depletion of MLL1 in ARPE-19 cells. Cells were transfected with siNS (non-specific siRNA) or siRNA targeting *MLL1* mRNA (siMLL1 or siMLL1(2)). After 72 h of transfection, total RNA was extracted by TRIzol and analyzed by RT-qPCR, and the mRNA levels were normalized to that of *18S* rRNA. Each value represents mean ± SD (*n* = 3), * *p* < 0.05. The total cell lysate was used to compare the protein level of MLL1 and β-actin by Western blot. (**B**) Enrichment of MLL1, RELA, H3K4me3, and Pol II at *HMOX1* promoter region determined by ChIP in ARPE-19 cells treated (+ marked) with A2E, BL, or A2E+BL. (**C**) Chromatin accessibility of *HMOX1* promoter. Formaldehyde-Assisted Isolation of Regulatory Elements (FAIRE)-qPCR (quantitative polymerase chain reaction) analysis was performed for the promoter region of *HMOX1*. Data were calculated as a percentage of input. Each value represents mean ± SD (*n* = 3), * *p* < 0.05.

MLL1 histone methylases contribute to the formation of chromatin structures suitable for transcriptional activation [44]. Therefore, we aimed to investigate the role of MLL1 in binding transcription factors and changes in chromatin structure during BL-induced *HMOX1* gene expression in ARPE-19 cells. First, two types of siRNAs (siMLL1 and siMLL1(2)), acting on different regions of *MLL1* mRNA, were used to reduce the level of *MLL1* expression in ARPE-19 cells (Figure 4A). Compared to cells treated with siNS, the occupancy of RELA in the promoter region of the *HMOX1* gene, which was increased by BL irradiation, was slightly suppressed in MLL1-depleted cells, but it was not statistically significant (Figure 4B). MLL1 histone methylase contributes to the formation of an open chromatin structure required for transcriptional activation by methylating the H3K4 residue [44]. Correspondingly, the level of H3K4me3 and the binding of RNA Pol II to the *HMOX1* promoter region were also significantly reduced by depletion of MLL1. Finally, we monitored changes in chromatin structure in the *HMOX1* promoter region following BL irradiation. BL irradiation significantly increased chromatin accessibility of the *HMOX1* promoter region, as expected. These changes are consistent with an increase in promoter recruitment of transcription factors, including NF-κB, and co-regulators, including MLL1. In contrast, depletion of MLL1 significantly inhibited the increase in chromatin accessibility induced by BL (Figure 4B,C). These results are consistent and show that MLL1 depletion significantly suppressed the induction of *HMOX1* expression by BL (Figure 3C,D). These results suggest that MLL1 plays an important role in H3K4me3 methylation in the *HMOX1* promoter region, NF-κB recruitment, and chromatin remodeling. 

### 3.5. Correlation of HMOX1 and MLL1 Expression Levels in AMD Patients

**Figure 5:**
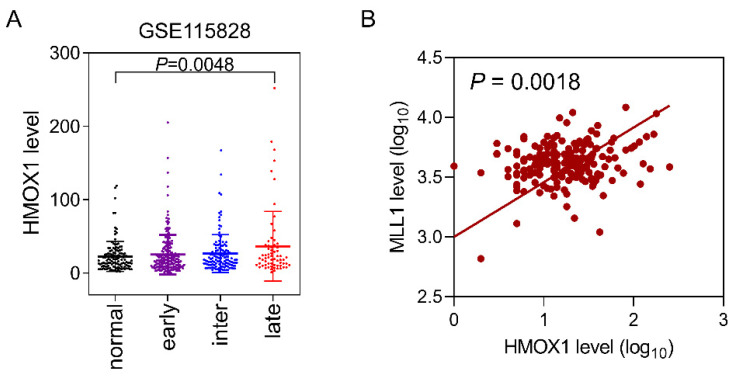
Correlation of *HMOX1* and *MLL1* expression levels in RPE cells of patients with dry AMD. (**A**) The mRNA levels of *HMOX1* in patients at different stages of dry AMD (GSE115828). Normal (*n* = 127), early (*n* = 197), intermediate (*n* = 126), and late (*n* = 67) stage AMD. (**B**) Correlation between *HMOX1* and *MLL1* mRNA levels in RPE cells from the patients with dry AMD (*n* = 47). Pearson correlation analysis of *MLL1* and *HMOX1* levels was conducted using the GraphPad Software Prism V.8.0.2.

In patients with dry macular degeneration, retinal drusen deposition is a clinical feature [45]. In addition, concerns regarding retinal damage caused by BL are increasing regardless of age because of the explosive increase in the use of smart devices. Therefore, we aimed to determine the clinical relevance of *HMOX1* expression in patients with dry AMD. AMD is classified into early-, intermediate-, and late-stage AMD, according to the pattern of drusen accumulation, and the formation of exudative neovascularization in the retina. To compare gene expression levels in the RPE cells of patients with dry AMD, a published transcriptome database (GSE115828) was used [46]. The expression of *HMOX1* in RPE cells showed a tendency to increase with symptom severity gradually. RPE cells from patients with late-stage AMD showed a statistically significant (*p* = 0.0048) increase in *HMOX1* expression compared to normal retinal tissues (Figure 5A). In addition, the expression levels of *MLL1* and *HMOX1* in retinal tissues showed a statistically significant correlation (Figure 5B). These results support our in vitro findings demonstrating that MLL1 regulates *HMOX1* expression in RPE cells.

## 4. Discussion

HMOX1 catalyzes oxidative degradation of heme groups. HMOX1 catalyzes the conversion of hemoglobin, heme, and myoglobin to bilirubin, and produces carbon monoxide (CO) and biliverdin as byproducts [47]. HMOX1 is generally expressed at a low level in all tissues and is remarkably induced in various stress environments, such as UV radiation, hydrogen peroxide, cytokines, hypoxia, and glutathione (GSH) consumption, which is thought to be a cellular defense mechanism [33]. Among the reaction byproducts, biliverdin is converted to bilirubin by biliverdin reductase. Bilirubin has strong antioxidant action and is responsible for the cellular protection mechanism of HMOX1 against oxidative stress.

Consistently, our results showed that HMOX1 is an important response factor for oxidative stress caused by A2E photooxidation in RPE cells. Through genome-wide transcriptome analysis, we showed that the photooxidation of A2E by BL activates the NF-κB pathway in ARPE-19 cells. Subsequent in silico analysis predicted RELA (p65) to be an upstream regulator of these genes. In addition, it was found that the expression of *HMOX1* was significantly increased by A2E photooxidation.

Among the major regulators of *HMOX1* transcription known to date, the transcription factor nuclear factor erythroid 2-related factor 2 (NRF2) and inducible repressor BTB domain and CNC homolog 1 (Bach1) appear to play the most important roles [48,49]. NRF2 is a basic leucine zipper protein that regulates the expression of antioxidant proteins in response to oxidative stress [50]. When cells are exposed to oxidative stress, activation of the NRF2/HMOX1 axis is induced through the c-Jun N-terminal kinase (JNK) and phosphoinositide-3 kinase (PI3K)-AKT pathways, resulting in the expression of various antioxidant proteins [51]. In addition to oxidative stress, stimulation of cytokines, such as IL-10 and IL-6 in immune cells, also induces the expression of *HMOX1*, which involves a signal transducer and transcription 3 activator (STAT3) [52,53]. In contrast to NRF2, which is recruited to the enhancer site located 4 and 10 kilobases upstream of the *HMOX1* gene [54,55], redox-sensitive transcription factors such as activator protein-1 (AP-1) and NF-κB bind to the promoter region of *HMOX1* to regulate transcription [56]. Under oxidative stress conditions, IκBα phosphorylated by IKK is released from the NF-κB dimer (p50/p65); as a result, NF-κB that migrates to the nucleus is recruited to the promoter site and induces *HMOX1* transcription [56]. 

Our ChIP assay results clearly showed a marked increase in the binding of RELA to the promoter region of *HMOX1* under BL. Notably, A2E itself did not affect *HMOX1* expression or the binding of RELA in the promoter region. Although there is likely a difference between the A2E concentration used in our study and the one present in vivo, BL significantly affected the physiological action of A2E in cells. These results suggest that exposure to blue light in daily life, and the degree of exposure to blue light, may act as more decisive factors in the damage of retinal cells than the accumulation of A2E with aging.

Next, we attempted to elucidate the function of MLL1 as a coactivator involved in the NF-κB-induced activation of *HMOX1* expression in ARPE-19 cells. NF-κB regulates gene expression through various post-translational modifications or modifications of histone molecules at target gene sites. For example, when Ser-276 of p65 (RELA) is phosphorylated by protein kinase A (PKA), a conformational change occurs in p65. Consequently, it promotes the binding of the cofactor cAMP response element binding protein (p300/CBP) at the target gene site [57]. CBP/P300 acetylates the N-terminus of histone molecules in chromatin and facilitates chromatin formation to increase transcriptional activity. In addition, the acetylation of Lys221 and Lys218 enhances the DNA binding of NF-κB (RELA), upregulating the expression of marker genes [58,59]

SET domain family proteins composed of SETD1A, SETD1B, and MLL1-MLL4 activate transcription by methylating histone H3K4 at the promoter or enhancer region of the gene [44]. In a previous report, the role of MLL1 in NF-κB target gene expression by TNFα stimulation in MEF cells was reported [43]. The functions of MLL1 in NF-κB activation in cancer cells are complex. In MLL-fused leukemia cells, the RELA-p50 complex binds to MLL1 and induces trimethylation of histone H3K4 residues in the promoter regions of *HOXA9* and *MEIS1*. In addition, the RELA-p50 complex recruits the MLL-fusion protein and the DOT1L protein that induces H3K79me2 functions in this process [60,61]. Our findings show, for the first time, the involvement of MLL1 in NF-κB activation by photooxidation stimulation of A2E in RPE cells. MLL1 helps trimethylation and chromatin accessibility of histone H3K4 in the promoter region of the *HMOX1*, and consequently facilitates the recruitment of RNA Pol II in RPE cells. In contrast, MLL2, which has a similar action as MLL1 on histone H3K4 methylation, does not have the same action as MLL1 on the expression of the *HMOX1*.

Functional impairment due to damage in RPE cells is one of the leading causes of AMD. In patients with early and intermediate AMD, called dry AMD, excessive accumulation of drusen components is characteristically observed in the retina. In contrast, some patients with late-stage AMD show the formation of new blood vessels called wet AMD [62]. The molecular biological pathogenesis of the dry AMD is not yet known, which limits the development of therapeutic agents. Recently, attempts have been made to protect RPE cells from oxidative stress by using natural products or low-molecular-weight compounds [26,27,28]. Another approach is to remove drusen accumulated in the retina using an intracellular degradation mechanism, such as autophagy [63]. Due to these efforts, the possibility of developing a therapeutic agent is gradually increasing. In any case, the need for a biomarker that correlates with the results of in vitro studies and AMD patients is becoming more important to predict desirable clinical outcomes. In this sense, our research results provide relevant alternatives. Our results suggest an important function of *HMOX1*, a well-known stress response gene, and MLL1, a coactivator, as novel biomarkers for AMD. The limitation of this study is the lack of experiments using primary RPE cells or animals. In order to overcome this and ensure in vivo correlation, we performed transcriptome analysis of clinical patients. Additionally, although our study suggested the differential function of MLL1 and MLL2 on HMOX1 gene expression, further investigation of the differential contribution of other H3K4 histone methyltransferases, such as SETD1A and SETD1B, is also needed.

## Data Availability

Not applicable.

## Supplementary Materials

The following supporting information can be downloaded at: https://www.mdpi.com/article/10.3390/life12111698/s1, Table S1: Primer sequences used for RT-qPCR; Table S2: Sequences of siRNAs

## References

[1] Taniguchi K., Karin M. (2018). NF-kappaB, inflammation, immunity and cancer: Coming of age. Nat. Rev. Immunol..

[2] Kaufman C.K., Fuchs E. (2000). It’s got you covered. NF-kappaB in the epidermis. J. Cell Biol..

[3] Adhami V.M., Afaq F., Ahmad N. (2003). Suppression of ultraviolet B exposure-mediated activation of NF-kappaB in normal human keratinocytes by resveratrol. Neoplasia.

[4] Devary Y., Rosette C., DiDonato J.A., Karin M. (1993). NF-kappa B activation by ultraviolet light not dependent on a nuclear signal. Science.

[5] Simon M.M., Aragane Y., Schwarz A., Luger T.A., Schwarz T. (1994). UVB light induces nuclear factor kappa B (NF kappa B) activity independently from chromosomal DNA damage in cell-free cytosolic extracts. J. Investig. Dermatol..

[6] O’Dea E.L., Kearns J.D., Hoffmann A. (2008). UV as an amplifier rather than inducer of NF-kappaB activity. Mol. Cell.

[7] Peak M.J., Peak J.G. (1990). Hydroxyl radical quenching agents protect against DNA breakage caused by both 365-nm UVA and by gamma radiation. Photochem. Photobiol..

[8] Cunningham M.L., Krinsky N.I., Giovanazzi S.M., Peak M.J. (1985). Superoxide anion is generated from cellular metabolites by solar radiation and its components. J. Free Radic. Biol. Med..

[9] Ryan T.C., Weil G.J., Newburger P.E., Haugland R., Simons E.R. (1990). Measurement of superoxide release in the phagovacuoles of immune complex-stimulated human neutrophils. J. Immunol. Methods.

[10] Morgan M.J., Liu Z.G. (2011). Crosstalk of reactive oxygen species and NF-kappaB signaling. Cell Res..

[11] Lingappan K. (2018). NF-kappaB in Oxidative Stress. Curr. Opin. Toxicol..

[12] Yoo J.A., Yu E., Park S.H., Oh S.W., Kwon K., Park S.J., Kim H., Yang S., Park J.Y., Cho J.Y. (2020). Blue Light Irradiation Induces Human Keratinocyte Cell Damage via Transient Receptor Potential Vanilloid 1 (TRPV1) Regulation. Oxidative Med. Cell. Longev..

[13] Sparrow J.R., Zhou J., Cai B. (2003). DNA is a target of the photodynamic effects elicited in A2E-laden RPE by blue-light illumination. Investig. Ophthalmol. Vis. Sci..

[14] Eldred G.E., Lasky M.R. (1993). Retinal age pigments generated by self-assembling lysosomotropic detergents. Nature.

[15] Eldred G.E., Katz M.L. (1988). Fluorophores of the human retinal pigment epithelium: Separation and spectral characterization. Exp. Eye Res..

[16] Parish C.A., Hashimoto M., Nakanishi K., Dillon J., Sparrow J. (1998). Isolation and one-step preparation of A2E and iso-A2E, fluorophores from human retinal pigment epithelium. Proc. Natl. Acad. Sci. USA.

[17] Holz F.G., Schutt F., Kopitz J., Eldred G.E., Kruse F.E., Volcker H.E., Cantz M. (1999). Inhibition of lysosomal degradative functions in RPE cells by a retinoid component of lipofuscin. Investig. Ophthalmol. Vis. Sci..

[18] Suter M., Reme C., Grimm C., Wenzel A., Jaattela M., Esser P., Kociok N., Leist M., Richter C. (2000). Age-related macular degeneration. The lipofusion component N-retinyl-N-retinylidene ethanolamine detaches proapoptotic proteins from mitochondria and induces apoptosis in mammalian retinal pigment epithelial cells. J. Biol. Chem..

[19] Sparrow J.R., Nakanishi K., Parish C.A. (2000). The lipofuscin fluorophore A2E mediates blue light-induced damage to retinal pigmented epithelial cells. Investig. Ophthalmol. Vis. Sci..

[20] Sparrow J.R., Zhou J., Ben-Shabat S., Vollmer H., Itagaki Y., Nakanishi K. (2002). Involvement of oxidative mechanisms in blue-light-induced damage to A2E-laden RPE. Investig. Ophthalmol. Vis. Sci..

[21] Ben-Shabat S., Itagaki Y., Jockusch S., Sparrow J.R., Turro N.J., Nakanishi K. (2002). Formation of a nonaoxirane from A2E, a lipofuscin fluorophore related to macular degeneration, and evidence of singlet oxygen involvement. Angew. Chem..

[22] Klionsky D.J., Petroni G., Amaravadi R.K., Baehrecke E.H., Ballabio A., Boya P., Bravo-San Pedro J.M., Cadwell K., Cecconi F., Choi A.M.K. (2021). Autophagy in major human diseases. EMBO J..

[23] Jeong S.Y., Gu X., Jeong K.W. (2019). Photoactivation of *N*-retinylidene-*N*-retinylethanolamine compromises autophagy in retinal pigmented epithelial cells. Food Chem. Toxicol..

[24] Jin H.L., Choung S.Y., Jeong K.W. (2017). Protective mechanisms of polyphenol-enriched fraction of *Vaccinium uliginosum* L. Against blue light-induced cell death of human retinal pigmented epithelial cells. J. Funct. Foods.

[25] Jin H.L., Jeong K.W. (2022). Transcriptome Analysis of Long-Term Exposure to Blue Light in Retinal Pigment Epithelial Cells. Biomol. Ther..

[26] Kim J., Jin H.L., Jang D.S., Jeong K.W., Choung S.Y. (2018). Quercetin-3-O-alpha-l-arabinopyranoside protects against retinal cell death via blue light-induced damage in human RPE cells and Balb-c mice. Food Funct..

[27] Pham T.N.M., Shin C.Y., Park S.H., Lee T.H., Ryu H.Y., Kim S.B., Auh K., Jeong K.W. (2021). *Solanum melongena* L. Extract Protects Retinal Pigment Epithelial Cells from Blue Light-Induced Phototoxicity in In Vitro and In Vivo Models. Nutrients.

[28] Shin C.Y., Lee M.H., Kim H.M., Chung H.C., Kim D.U., Lee J.H., Jeong K.W. (2022). Protective Effect of *Ribes nigrum* Extract against Blue Light-Induced Retinal Degeneration In Vitro and In Vivo. Antioxidants.

[29] Xie Z., Bailey A., Kuleshov M.V., Clarke D.J.B., Evangelista J.E., Jenkins S.L., Lachmann A., Wojciechowicz M.L., Kropiwnicki E., Jagodnik K.M. (2021). Gene Set Knowledge Discovery with Enrichr. Curr. Protoc..

[30] Kuleshov M.V., Jones M.R., Rouillard A.D., Fernandez N.F., Duan Q., Wang Z., Koplev S., Jenkins S.L., Jagodnik K.M., Lachmann A. (2016). Enrichr: A comprehensive gene set enrichment analysis web server 2016 update. Nucleic Acids Res..

[31] Jin M.L., Yang L., Jeong K.W. (2022). SETD1A-SOX2 axis is involved in tamoxifen resistance in estrogen receptor alpha-positive breast cancer cells. Theranostics.

[32] Chen X., Kandasamy K., Srivastava R.K. (2003). Differential roles of RelA (p65) and c-Rel subunits of nuclear factor kappa B in tumor necrosis factor-related apoptosis-inducing ligand signaling. Cancer Res..

[33] Sebastian V.P., Salazar G.A., Coronado-Arrazola I., Schultz B.M., Vallejos O.P., Berkowitz L., Alvarez-Lobos M.M., Riedel C.A., Kalergis A.M., Bueno S.M. (2018). Heme Oxygenase-1 as a Modulator of Intestinal Inflammation Development and Progression. Front. Immunol..

[34] Datta S., Cano M., Ebrahimi K., Wang L., Handa J.T. (2017). The impact of oxidative stress and inflammation on RPE degeneration in non-neovascular AMD. Prog. Retin. Eye Res..

[35] Sanvicens N., Gomez-Vicente V., Masip I., Messeguer A., Cotter T.G. (2004). Oxidative stress-induced apoptosis in retinal photoreceptor cells is mediated by calpains and caspases and blocked by the oxygen radical scavenger CR-6. J. Biol. Chem..

[36] Sparrow J.R., Vollmer-Snarr H.R., Zhou J., Jang Y.P., Jockusch S., Itagaki Y., Nakanishi K. (2003). A2E-epoxides damage DNA in retinal pigment epithelial cells. Vitamin E and other antioxidants inhibit A2E-epoxide formation. J. Biol. Chem..

[37] Donato L., Scimone C., Alibrandi S., Pitruzzella A., Scalia F., D’Angelo R., Sidoti A. (2020). Possible A2E Mutagenic Effects on RPE Mitochondrial DNA from Innovative RNA-Seq Bioinformatics Pipeline. Antioxidants.

[38] Kang C., Xu Q., Martin T.D., Li M.Z., Demaria M., Aron L., Lu T., Yankner B.A., Campisi J., Elledge S.J. (2015). The DNA damage response induces inflammation and senescence by inhibiting autophagy of GATA4. Science.

[39] Kuilman T., Michaloglou C., Vredeveld L.C., Douma S., van Doorn R., Desmet C.J., Aarden L.A., Mooi W.J., Peeper D.S. (2008). Oncogene-induced senescence relayed by an interleukin-dependent inflammatory network. Cell.

[40] Chien Y., Scuoppo C., Wang X., Fang X., Balgley B., Bolden J.E., Premsrirut P., Luo W., Chicas A., Lee C.S. (2011). Control of the senescence-associated secretory phenotype by NF-kappaB promotes senescence and enhances chemosensitivity. Genes Dev..

[41] Freund A., Patil C.K., Campisi J. (2011). p38MAPK is a novel DNA damage response-independent regulator of the senescence-associated secretory phenotype. EMBO J..

[42] Wang P., Han L., Shen H., Wang P., Lv C., Zhao G., Niu J., Xue L., Wang Q.J., Tong T. (2014). Protein kinase D1 is essential for Ras-induced senescence and tumor suppression by regulating senescence-associated inflammation. Proc. Natl. Acad. Sci. USA.

[43] Wang X., Zhu K., Li S., Liao Y., Du R., Zhang X., Shu H.B., Guo A.Y., Li L., Wu M. (2012). MLL1, a H3K4 methyltransferase, regulates the TNFalpha-stimulated activation of genes downstream of NF-kappaB. J. Cell Sci..

[44] Shilatifard A. (2008). Molecular implementation and physiological roles for histone H3 lysine 4 (H3K4) methylation. Curr. Opin. Cell Biol..

[45] Buschini E., Piras A., Nuzzi R., Vercelli A. (2011). Age related macular degeneration and drusen: Neuroinflammation in the retina. Prog. Neurobiol..

[46] Ratnapriya R., Sosina O.A., Starostik M.R., Kwicklis M., Kapphahn R.J., Fritsche L.G., Walton A., Arvanitis M., Gieser L., Pietraszkiewicz A. (2019). Retinal transcriptome and eQTL analyses identify genes associated with age-related macular degeneration. Nat. Genet..

[47] Ryter S.W., Alam J., Choi A.M. (2006). Heme oxygenase-1/carbon monoxide: From basic science to therapeutic applications. Physiol. Rev..

[48] Alam J., Stewart D., Touchard C., Boinapally S., Choi A.M., Cook J.L. (1999). Nrf2, a Cap’n’Collar transcription factor, regulates induction of the heme oxygenase-1 gene. J. Biol. Chem..

[49] Sun J., Hoshino H., Takaku K., Nakajima O., Muto A., Suzuki H., Tashiro S., Takahashi S., Shibahara S., Alam J. (2002). Hemoprotein Bach1 regulates enhancer availability of heme oxygenase-1 gene. EMBO J..

[50] Chen H.H., Chen Y.T., Huang Y.W., Tsai H.J., Kuo C.C. (2012). 4-Ketopinoresinol, a novel naturally occurring ARE activator, induces the Nrf2/HO-1 axis and protects against oxidative stress-induced cell injury via activation of PI3K/AKT signaling. Free. Radic. Biol. Med..

[51] Xiao Q., Piao R., Wang H., Li C., Song L. (2018). Orientin-mediated Nrf2/HO-1 signal alleviates H2O2-induced oxidative damage via induction of JNK and PI3K/AKT activation. Int. J. Biol. Macromol..

[52] Gomez-Hurtado I., Zapater P., Bellot P., Pascual S., Perez-Mateo M., Such J., Frances R. (2011). Interleukin-10-mediated heme oxygenase 1-induced underlying mechanism in inflammatory down-regulation by norfloxacin in cirrhosis. Hepatology.

[53] Ricchetti G.A., Williams L.M., Foxwell B.M. (2004). Heme oxygenase 1 expression induced by IL-10 requires STAT-3 and phosphoinositol-3 kinase and is inhibited by lipopolysaccharide. J. Leukoc. Biol..

[54] Alam J., Cook J.L. (2007). How many transcription factors does it take to turn on the heme oxygenase-1 gene?. Am. J. Respir. Cell Mol. Biol..

[55] Balogun E., Hoque M., Gong P., Killeen E., Green C.J., Foresti R., Alam J., Motterlini R. (2003). Curcumin activates the haem oxygenase-1 gene via regulation of Nrf2 and the antioxidant-responsive element. Biochem. J..

[56] Paine A., Eiz-Vesper B., Blasczyk R., Immenschuh S. (2010). Signaling to heme oxygenase-1 and its anti-inflammatory therapeutic potential. Biochem. Pharmacol..

[57] Zhong H., SuYang H., Erdjument-Bromage H., Tempst P., Ghosh S. (1997). The transcriptional activity of NF-kappaB is regulated by the IkappaB-associated PKAc subunit through a cyclic AMP-independent mechanism. Cell.

[58] Chen L.F., Mu Y., Greene W.C. (2002). Acetylation of RelA at discrete sites regulates distinct nuclear functions of NF-kappaB. EMBO J..

[59] Kiernan R., Bres V., Ng R.W., Coudart M.P., El Messaoudi S., Sardet C., Jin D.Y., Emiliani S., Benkirane M. (2003). Post-activation turn-off of NF-kappa B-dependent transcription is regulated by acetylation of p65. J. Biol. Chem..

[60] Goyama S., Mulloy J.C. (2013). NF-kappaB: A coordinator for epigenetic regulation by MLL. Cancer Cell.

[61] Kuo H.P., Wang Z., Lee D.F., Iwasaki M., Duque-Afonso J., Wong S.H., Lin C.H., Figueroa M.E., Su J., Lemischka I.R. (2013). Epigenetic roles of MLL oncoproteins are dependent on NF-kappaB. Cancer Cell.

[62] Ferris F.L., Fine S.L., Hyman L. (1984). Age-related macular degeneration and blindness due to neovascular maculopathy. Arch. Ophthalmol..

[63] Zhang Q., Presswalla F., Ali R.R., Zacks D.N., Thompson D.A., Miller J.M.L. (2021). Pharmacologic activation of autophagy without direct mTOR inhibition as a therapeutic strategy for treating dry macular degeneration. Aging.

