# Supplementary material for: Photooxidation of A2E by Blue Light Regulates Heme Oxygenase 1 Expression via NF-κB and Lysine Methyltransferase 2A in ARPE-19 Cells
Source: Life (Basel). 2022 Oct 25;12(11):1698. doi: 10.3390/life12111698 (PMC9699413; doi:10.3390/life12111698)
Supplement: Supplementary file 1 [file life-12-01698-s001.zip › life-1967902-supplementary.pdf]

Supplementary Materials

Supplementary Table S1. Primer sequences used for RT-qPCR

Gene	Forward	Reverse
HMOX1	CCTGGAAGACACCCTAATG	AAACACAAGACACAACATCT
HSP90AA1	GTTGGTCCTGTGCGGTCACT	TGGGCAATTTCTGCCTGAA
DUSP5	GTCCTCACCTCGCTACTC	GGGCTCTCTCACTCTCAAT
INHBA	TGTGATGGCAAGGTCAACT	ATGATAGCCAGAGGGAGCAA
CXCL8	CTGGCCGTGGCTCTCTTG	CCTTGGCAAAACTGCACCTT
TRIB1	TTCAAGCAGATTGTCTCC GC	AGTGGTGTTGAGGATCTCAG
MLL1	GAGGACCCCGGATTAAACAT	GGAGCAAGAGGTTCAGCATC
MLL2	GTGCAGCAGAAGATGGTGAA	GCACAATGCTGTCAGGAGAA
HMOX1 promoter (–250 bp)	CAGAGCCTGCAGCTTCTCAGA	GGAAACAAAGTCTGGCCATAGGAC
HMOX1 enhancer (– 4 kb)	CAGTGCCTCCTCAGCTTCTC	CTCGGTGGATTGCAACATTA
18S	GAGGATGAGGTGGAACGTGT	TCTTCAGTCGCTCCAGGTCT

Supplementary Table S2. Sequences of siRNAs

Name	Sequence
siNS	UUCUCCGAACGUGUCACGUdTdT (sense)<br>ACGUGACACGUUCGGAGAAdTdT (anti-sense)
siMLL1	GAUUCGAACACCCAGUUAUdTdT (sense)<br>AUAACUGGGUGUUCGAAUCdTdT (anti-sense)
siMLL1(2)	GCACUGUUAAACAUUCCACdTdT (sense)<br>GUGGAAUGUUUAACAGUGCdTdT (anti-sense)
siMLL2	CCCACCUGAAUCAUCACCUdTdT (sense)<br>AGGCGAUGAUUCAGGUGGGdTdT (anti-sense)
